# Extracorporeal shock wave therapy in the supportive care and rehabilitation of cancer patients

**DOI:** 10.1007/s00520-019-05046-y

**Published:** 2019-08-24

**Authors:** Richard Crevenna, Michael Mickel, Mohammad Keilani

**Affiliations:** Department of Physical Medicine, Rehabilitation and Occupational Medicine, Medical University of Vienna, Waehringer Guertel 18-20, A-1090 Vienna, Austria

**Keywords:** Cancer survivors, Shock wave treatment, Musculoskeletal, Supportive care, Rehabilitation

## Abstract

**Purpose:**

Cancer patients sometimes show immobilizing musculoskeletal conditions which prohibit active exercise due to severe bodily pain. Therefore, before starting a rehabilitative exercise program, the pain has to be reduced to enable the patient to participate actively in the exercise program. Extracorporeal shock wave therapy (ESWT, the application of radial and/or focused shock waves with low or high energy) has been shown to be effective and efficient in the treatment of musculoskeletal disorders. However, one historical paradigm was the fact that, in the past, cancer was seen as a contraindication for the use of ESWT.

**Methods:**

Clinical note to present indications, benefits, and contraindications of shock wave treatment in cancer patients.

**Results:**

Malignant tumors in the treatment area have to be seen as a contraindication for the use of ESWT treatment. Cancer itself—in the form of the underlying disease—is not a contraindication for the treatment with radial and focused shock wave therapy with low or high energy. Plantar fasciitis and calcaneal spurs, calcified shoulder, tennis elbow or Achilles tendinopathy, and delayed healing and chronic wounds are typical approved standard indications for ESWT, and are allowed when the malignant tumor is not in the treatment area. There are also other musculoskeletal and non-musculoskeletal indications (e.g., myofascial syndrome, erectile dysfunction, polyneuropathy, and lymphedema) that are relevant for cancer survivors. These indications are recommended by the International Society for Medical Shockwave Treatment (ISMST) for “common empirically tested clinical use” and as exceptional indications/expert indications.

**Conclusion:**

ESWT is a safe and relevant modality for the supportive care and rehabilitation of cancer patients.

## Commentary

Cancer rehabilitation has been shown to be a relevant means to accelerate return to work and improve the work ability and social participation of cancer survivors [1–3]. In cancer rehabilitation, physical modalities such as exercise to improve muscular strength, endurance capacity, sensorimotor functions, and flexibility are of a very high relevance [3]. Nevertheless, some musculoskeletal conditions, such as plantar fasciitis or calcaneal spurs, calcifying tendinopathy of the shoulder, and tennis elbow or Achilles tendinopathy, immobilize the patient and prohibit active participation in exercise, due to severe bodily pain [1, 2]. Therefore, before starting a rehabilitative exercise program, the bodily pain has to be reduced to enable the patient to participate actively [1, 2].

Extracorporeal shock wave therapy (ESWT) in musculoskeletal disorders is a conservative treatment modality, which has been developed over the last 25 years, and has been shown to be both very effective and time- and cost-efficient [2, 4]. Nevertheless, there are several historical (and “traditional”) paradigms which have changed during this time period; one of them was the fact that, in the past, cancer represented a contraindication for the use of ESWT [1, 2, 5].

The International Society for Medical Shockwave Treatment (ISMST) (the managing board, the advisory board and the Senators of the ISMST) issued clinical recommendations for the use of therapeutic shock waves in clinical practice in October 2016 [4].

These recommendations were assembled based on an assessment of the current published scientific and clinical information and accepted approaches to treatment, and were intended to aid the clinician in the use of ESWT [4]. In particular, the guidelines were intended to clarify the indications and contraindications for treatment [4]. Malignant tumors, metastasis, multiple myeloma, and lymphoma in the treatment area have to be seen as contraindications for treatment with radial and focused shock waves with low and high energy. Cancer itself, in the form of the underlying disease, is not a contraindication for ESWT [4]. Active leukemia and leukemic phase of lymphoma (not in remission) are, however, contraindications for the use of ESWT. A minimum level of standard examinations before performing ESWT is necessary, including clinical examination, radiological imaging, and neurological and/or laboratory diagnostic tests.

Only a qualified physician should use focused shock wave therapy to treat pathologies which have been determined by diagnostic testing [4].

For ESWT, there are approved standard indications (for example, plantar fasciitis, with or without heel spur; calcifying tendinopathy of the shoulder; lateral epicondylopathy of the elbow/tennis elbow or Achilles tendinopathy; bone non-union/pseudarthroses; delayed or non-healing wounds), common empirically tested clinical uses (for example, bone marrow edema, different tendinopathies, myofascial syndrome), and so-called “exceptional indications” (expert indications, for example, erectile dysfunction, polyneuropathy, and lymphedema) [4].

In cancer rehabilitation, there are typical side-effects to overcome, such as polyneuropathy, lymphedema, musculoskeletal pain, and reduced physical performance. These side-effects need effective and, in most cases, time-efficient treatment. The interdisciplinary field of physical medicine and rehabilitation (PM&R) has its clinical research focus on the treatment of musculoskeletal pain, polyneuropathy, and lymphedema, and on an improvement of physical performance by the use of exercise [1]. At our department, we have a strong expertise in the use of ESWT for different conditions such as calcaneal spurs, calcific tendinitis of the shoulder, tennis elbow, and musculoskeletal pain syndromes. Secondary complications of cancer and its treatment, such as lymphedema and polyneuropathy, are of great relevance for many cancer patients. Since a specific causal therapy for those disorders does not exist, they are currently the focus of our research [1, 4, 6].

ESWT as a physical treatment option is only contraindicated at the tumor site. Unfortunately, many physicians still deem cancer as a general contraindication for the use of ESWT. As a result, many cancer patients remain undertreated and, in the worst cases, cannot be mobilized due to bodily pain [2]. There are, however, additional conditions (e.g., polyneuropathy or lymphedema) for the use of ESWT in cancer patients recommended by the ISMST as exceptional indications or expert indications [4, 7, 8]. For these indications (polyneuropathy and lymphedema), in our opinion, a superficial area-wide ESWT treatment (not at the tumor site) of the affected limb is suggested.

Erectile dysfunction is another condition of interest in cancer rehabilitation [9–11]. This indication can also be treated with ESWT, but has to be managed in an interdisciplinary setting [2].

In our opinion, ESWT is a very effective, safe, and time- and cost-efficient method, which can be considered as an interesting modality in the supportive care and rehabilitation of cancer patients. ESWT could thus be an effective way to reduce pain and mobilize cancer patients to attend rehabilitation programs and/or to return to work, for example, in cases of calcaneal spurs or calcific tendinitis of the shoulder, which are both very immobilizing conditions in both cancer and non-cancer patients. Thus, ESWT can be considered as a good measure when being used for the above-described indications. Nevertheless, further research is urgently needed to identify the technical parameters (number of sessions, energy transmitted) to implement ESWT as a safe and efficient tool for specific indications (e.g., polyneuropathy or lymphedema) in cancer rehabilitation.

## References

[1] Crevenna R, Kainberger F, Wiltschke C, Marosi C, Wolzt M, Cenik F, Keilani M (2018) Cancer rehabilitation: current trends and practices within an Austrian University Hospital Center. Disabil Rehabil:1–6. 10.1080/09638288.2018.1514665 10.1080/09638288.2018.1514665 30328719

[2] Crevenna R (2018) Physikalische Medizin und Rehabilitation: ein Kurzlehrbuch. Facultas Wien

[3] Cenik F, Mähr B, Palma S, Keilani M, Nowotny T, Crevenna R The role of physical medicine for cancer rehabilitation and return to work under the premise of the “Wiedereingliederungsteilzeitgesetz”. Wien Klin Wochenschr accepted/in press, WKWO-D-19-00044 10.1007/s00508-019-1504-7 PMC6795628 31087151

[4] https://www.shockwavetherapy.org/fileadmin/user_upload/dokumente/PDFs/Formulare/ISMST_consensus_statement_on_indications_and_contraindications_20161012_final.pdf (on 27th of April 2019)

[5] Lohrer H, Nauck T, Korakakis V, Malliaropoulos N (2016). Historical ESWT paradigms are overcome: a narrative review. Biomed Res Int.

[6] Crevenna R, Ashbury FD (2018). Physical interventions for patients suffering from chemotherapy-induced polyneuropathy. Support Care Cancer.

[7] Lohse-Busch H, Marlinghaus E, Reime U, Möwis U (2014). Focused low-energy extracorporeal shock waves with distally symmetric polyneuropathy (DSPNP): a pilot study. NeuroRehabilitation..

[8] Schaupper M, Jeltsch M, Rohringer S, Redl H, Holnthoner W (2016). Lymphatic vessels in regenerative medicine and tissue engineering. Tissue Eng Part B Rev.

[9] Dong L, Chang D, Zhang X, Li J, Yang F, Tan K, Yang Y, Yong S, Yu X (2019). Effect of low-intensity extracorporeal shock wave on the treatment of erectile dysfunction: a systematic review and meta-analysis. Am J Mens Health.

[10] Sokolakis I, Hatzichristodoulou G (2019). Clinical studies on low intensity extracorporeal shockwave therapy for erectile dysfunction: a systematic review and meta-analysis of randomised controlled trials. Int J Impot Res.

[11] Campbell JD, Trock BJ, Oppenheim AR, Anusionwu I, Gor RA, Burnett AL (2019). Meta-analysis of randomized controlled trials that assess the efficacy of low-intensity shockwave therapy for the treatment of erectile dysfunction. Ther Adv Urol.

